# Materials for the study of the *locus operandi* in the search for missing persons in Italy

**DOI:** 10.1080/20961790.2020.1854501

**Published:** 2021-02-23

**Authors:** Pier Matteo Barone, Rosa Maria Di Maggio, Silvia Mesturini

**Affiliations:** a Archaeology and Classics Program, American University of Rome, Rome, Italy; b Geoscienze Forensi Italia^®^ -Forensic Geoscience, Rome, Italy; c Comitato Scientifico Ricerca Scomparsi, Rome, Italy; d Studio Legale e Criminologico Mesturini, Livorno, Italy

**Keywords:** Forensic sciences, geographic profiling, *locus operandi*, geographic information systems, missing persons

## Abstract

Geographic profiling, or *locus operandi*, is a scientific approach that has been applied to forensic investigations for several years. However, it has never been applied to a phenomenon as complex as the search for missing persons. This article presents three Italian case studies in which geographic profiling was used to narrow the areas to search for missing persons. The geographical data were mapped and processed on a geographic information system platform using computational geometry analysis. However, these disappearances occurred during the Italian lockdown period, imposed to address the COVID-19 outbreak, which made the searches slower and more complex than usual.

## Introduction

Geographic information systems (GIS) are trusted and widely used in the field of forensic science to obtain geo-databases that can be used for investigation. The goal of this paper is to develop the use of GIS in forensic research beyond mapping, by using its spatial analytical capabilities, to offer a series of decision-making support tools. This allows better management and understanding of the complex and interconnected nature of forensic research on the ground [1–13]. It can also be applied to counter a phenomenon that has unfortunately become a social scourge in Italy and abroad: missing persons.

Searching for missing persons is a complex process, particularly in a country like Italy, where 61 036 people disappeared between 1974 and 2019. The position of Special Commissioner for missing persons was established by the Italian Government at the Ministry of the Interior in July 2007. The Commissioner’s 22nd report for 2019 highlighted the enormity of the underlying problem. In 2019 alone, 15 044 denunciations were filed in Italy pertaining to missing persons. Although fewer than 2018 with a total of 18 393 denunciations, the numbers remain significant [14]. The Commissioner’s report culminated in a presidential decree, motivated by the need for specific coordination to research and monitor the phenomenon. According to the decree, territorial prefectures are responsible for adapting and updating provincial plans for searching for missing persons and following national and regional laws, the guidelines issued by the Special Commissioner, and the circulars of the Ministry of the Interior [15]. This means that it is not necessary for a missing person to have been involved in a criminal event (e.g. becoming a victim of kidnapping or child abduction, or escaping from judicial authorities) but simply to have disappeared (e.g. to escape domestic abuse or problems, to live somewhere else under a new identity, to commit suicide, because of mental illness, to abandon their current social situation, because of financial loss, or to take any kind of advantage). In these latter cases, tracing the missing person is more complicated. The creation of provincial plans for missing persons is intended to define the organisational structure, operational roles, and activities related to searching and guaranteeing the maximum possible effectiveness of the rescue device. This must take account of the extreme morphological diversity of the country; depending on the case, the involvement of many research groups, specialising in different fields, may be required [16].

All over the world, forensic research systems have rapidly improved with the use of new technologies [17, 18], but the use of digital approaches is still underused. In particular, what is known as geographic profiling in English (or *locus operandi* in Latin) could be a more promising approach. Although this technique is known in forensic circles as a method for crime prevention [19], it has never yet been applied in the search for missing persons.

Rossmo [20] created this approach and developed the following formula:

(1) $$p_{i,j}=k\sum_{n=1}^{\left(\mathrm{total crimes}\right)}\left[\frac{\phi_{ij}}{{\left(\left|X_{i}-x_{n}|+|Y_{j}-y_{n}\right|\right)}^{f}}+\frac{\left(1-\phi_{ij})(B^{g-f}\right)}{{\left(2B-|X_{i}-x_{n}|-|Y_{j}-y_{n}|\right)}^{g}}\right]$$

where

ϕij={1, se(|Xi−xn|+|Yj−yn|)>B0, otherwise

Despite its complex appearance, the formula in Equation (1) is relatively simple to understand. Consider a map with an overlapping grid of squares, called sectors. If this map is an image file, called a *raster*, these sectors are *pixels*. A sector $S_{i,j}$ is the square on row *i* and column *j*, with coordinates ($X_{i},$$Y_{j}$). Equation (1) defines the probability $p_{i,j}$ of the criminal’s location being within a specific sector ($X_{i},$$Y_{j}$). A sum is computed for the previous crimes, with coordinates ($x_{n},$$y_{n}$) for the location of each crime. $\phi_{ij}$ is the characteristic function whose value is 0 when the point ($X_{i},$$Y_{j}$) (the residence of the wanted criminal) is within a distance of B from the crime location. $\phi_{ij}$ allows the definition of p to switch from one term to the other. If a crime occurs within this *buffer zone*, then $\phi_{ij}=0$ and therefore the first term does not contribute to the overall outcome. This ($\phi_{ij}=1$) is a prerequisite for defining the first term, preventing the distance between two points (or pixels) becoming equal to zero. When $\phi_{ij}=1,$ the first term is used to calculate $p_{i,j}.$ $\left|X_{i}-x_{n}|+|Y_{j}-y_{n}\right|$ is the so-called Manhattan distance (or taxicab metric) [21] between the point ($X_{i},$$Y_{j}$) and the *n*th crime site ($x_{n},$$y_{n}$) [19].

The sum in Equation (1) comprises two terms. The first term describes the decreasing probability of a crime with increasing distance. The second term refers to the concept of a buffer zone. The function ϕ is used to select one of the two concepts. The variable B describes the radius of the buffer zone, and the constant k is determined empirically. The main meaning of the equation is that the probability of a crime increases at first as the location moves within the buffer zone, away from the hot zone (the criminal’s location), but then decreases with distance outside the buffer zone. The variable f can be chosen to work best on data from previous crimes. The same principle applies to the variable g. The distance is then calculated with the formula for Manhattan distance [22, 23]. Rossmo’s formula marked the beginning of the usage and popularity of geographic profiling [23, 24].

In this article, a conceptually similar, but technically different approach will be applied. Geographic profiling is proposed as a logistical and concrete support to law enforcement agencies searching for missing persons that are not related to a crime. Some case studies will help to demonstrate the effectiveness of this methodology.

A location affected by a disappearance or crime reveals the dynamics of the event as well as the spatial behaviour of both the victim and the offender. The spatial analysis of crime sites is relevant in the context of urban security, through the analysis of crime hotspots. However, it can also provide useful information for tracing the possible areas of residence of both the victim and criminal, whose data are merged in geographic profiling.

Geographic profiling is an investigative procedure that can be applied to disappearances, kidnappings, serial killings, serial rapes, serial arson attacks, bomb attacks, and bank robberies. It helps study the places affected by the victim and the criminal and delimits a geographical area as a possible location for both the known victim and the unknown offender. The method shifts the investigation to the geographic data of the places where the offence occurred and provides the analyst with a “priority area”, in which investigative resources can be invested and managed better. Moreover, it provides general indications about the movement patterns of both the victim and the offender in the geographical space.

### Data acquisition and processing

With some adjustment, geographic profiling can be applied to a search for missing persons immediately upon reception of a missing person report. By focusing on the habits of the missing person, a search party can evaluate the places frequented by the person and statistically hypothesise the area in which the person may be found. This approach has the potential to drastically and effectively reduce search areas to a few hundred square metres, whereas normally many hectares are covered, often with negative results.

The principle is similar to that of traditional geographic profiling. However, instead of focusing on criminals and their hot zones, we analyse the areas frequented by the missing person before their disappearance because no crimes are supposed to be committed at the first step.

Adapting Rossmo’s formula to the missing person scenario, the sectors $S_{i,j}$ with coordinates ($X_{i},$$Y_{j}$) indicate the most frequented places. Equation (1) defines the probability $p_{i,j}$ of the missing person’s location being within a specific sector ($X_{i},$$Y_{j}$). A sum is computed for the previous frequented places, with coordinates ($x_{n},$$y_{n}$) for the location of each previously frequented place. $\phi_{ij}$ is the characteristic function whose value is 0 when the point ($X_{i},$$Y_{j}$) (the residence of the missing person) is within a distance of B from the previous frequented place. $\phi_{ij}$ allows the definition of p to switch from one term to the other. If a disappearance occurs within the *buffer zone*, then $\phi_{ij}=0$ and therefore the first term does not contribute to the overall outcome. Here, as in Rossmo’s formula, the sum comprises two terms. The first term describes the decreasing probability of finding the person with increasing distance. The second term refers to the concept of a buffer zone. The function ϕ is used to select one of the two concepts. The variable B describes the radius of the buffer zone. The constant k is determined empirically, based on how long a place was frequented by the missing person before disappearance. The main meaning of the equation is that the probability to find a missing person increases at first as the location moves within the buffer zone, away from the hot zone (the missing person’s previous frequented locations), but then decreases with distance outside the buffer zone.

To increase the potential of this application of the equation, and for more effective geographic profiling, geographical data must be processed using computational geometry. Computational geometry is the branch of geometry that studies efficient algorithms for solving geometric problems, and their computer implementations [25, 26]. The following paragraphs explain the three computational analyses used during the creation of the geographic profile. The superimposition of these computational vectorial layers, together with the information derived from the adapted Rossmo formula, defines a search area from which the police can start.

### Thiessen polygons

Thiessen polygons are an essential method for proximity analysis and are used to associate each position in space with its closest point. This space is defined as the area around a point in which every position is closer to that point than to any other. These types of structures can also be generated in higher dimensions, and these are called Thiessen polyhedra [27, 28].

The possible applications for Thiessen polygons are varied, as a consequence of their organisation, which is similar to many phenomena observed in geosciences and nature (such as plant cells and soap bubbles colliding with each other). They are used to generate soil maps based on irregularly distributed sample points. The boundary between two soil types is assumed to be the half-way point between two sample points with differing soil types. No further information about the space is assumed [29].

Thiessen polygons can be constructed by a geometric approach. A Thiessen polygon encloses all the space that is closer to the associated central point than to any other. It is obvious that the boundaries between two Thiessen polygons are lines that are equidistant from two central points. To construct the Thiessen polygons, all points are triangulated in an irregular triangular network. For each edge of each triangle, perpendicular bisectors are generated, which form the edges of the Thiessen polygons. Perpendicular bisectors are constructed by drawing circles that radiate from the corresponding points. The vertices of the Thiessen polygon are located at the points where the bisectors intersect. By superimposing the vector result on a raster satellite image, the areas inside the polygons show the positions closest to a certain point. Raster data models have advantages over vector data models because it is possible to choose the metric space and include weighting factors in the calculation [30].

### Voronoi diagrams

In general, Voronoi diagrams decompose a set of objects within the same space into a set of polygonal partitions. They help to illustrate the proximity and distance of the objects (i.e. a points) placed in a separate polygon. A polygonal shape surrounds each object in such a way that nearly every point of the polygon is closer to its generated object than to any other generated object, for any set of objects, in either a two-dimensional (2 D) or three-dimensional space. Unlike the Thiessen polygons, to which it is sometimes compared, a Voronoi diagram can also be created around lines, which leads to more complex structures [31, 32].

### Delaunay triangulations

**Figure 1.**
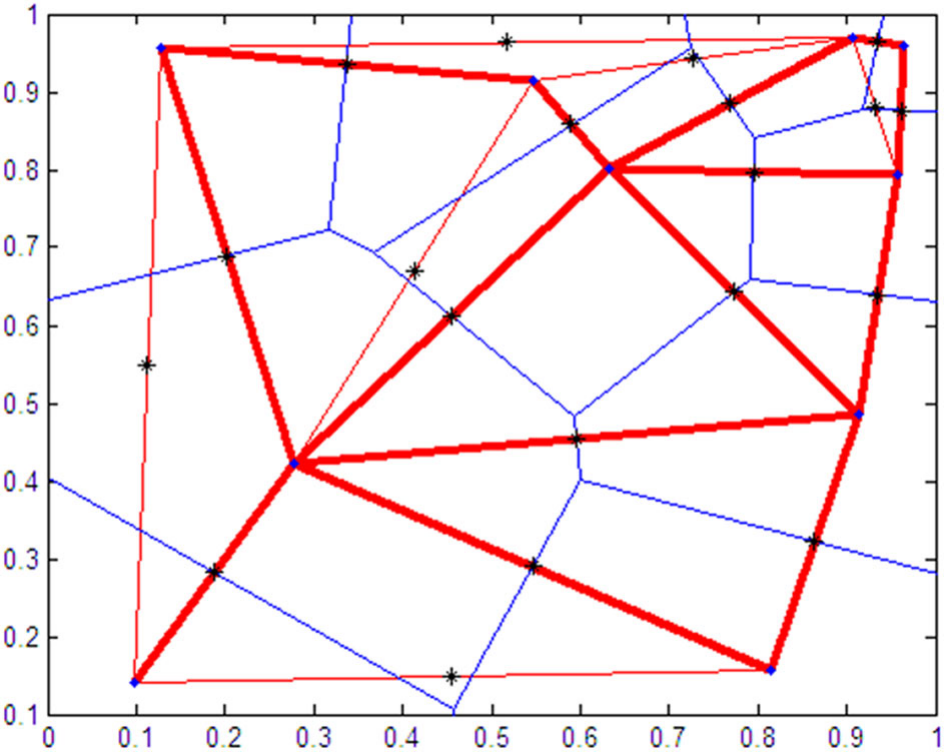
The area enclosed by red thin lines shows how the overlapping Thiessen (black asterisks), Voronoi (blue lines), and Delaunay (red thick lines) models lead to the circumscription of a specific area.

Delaunay triangulations are widely used in scientific computing for many different applications. Although there are numerous algorithms for computational triangulation, the favourable geometric properties of Delaunay triangulation make it very useful [33]. The fundamental property is the Delaunay criterion. In the case of 2 D triangulations, this is often called the empty circumference criterion. For a set of 2 D points, in a Delaunay triangulation, the circumference associated with each triangle does not contain other points within it. The Delaunay triangulation for a set of points generates triangles that connect all the points so that each triangle has no points in its circumference other than the three corners. Therefore, after the triangulation of all points, the Delaunay triangulation has the maximum minimum angle. That is, if the minimum angle of all of the triangles is considered, that angle in the Delaunay triangulation will be the largest of the minimum angles in all triangulations. The Delaunay triangulation is the dual structure of the Voronoi diagram [34, 35], and together they help to restrict the search area (Figure 1).

Statistical validation is required for the verification of the result. In particular, the use of geostatistics allows the spatial autocorrelation of the data to be assessed, and can verify whether observations made at nearby points actually have a greater correlation than observations made at distant points. Therefore, the aim is to assess how this autocorrelation varies according to the separation vector considered (distance and direction) [36]. Geostatistics—a sub-discipline of spatial statistics—has been developed over the past three decades and is well-established and widely applied in environmental research and technology. It includes a set of statistical methods concerning random variables with spatial or temporal variability (random fields). These variables represent physical quantities of economic or environmental importance. The methods are based on the assumption that space–time variability includes a random component that has a space–time correlation [4, 11, 13]. Therefore, statistical measures such as mean, variance, standard deviation, and spatiotemporal dependence are used to extract useful information from the available data. Geostatistics deals with distributions in which spatial or temporal dependence is the primary characteristic. Geostatistical analysis aims to estimate the statistical parameters that determine the spatial or temporal distribution and the dependence of the relevant variables. This procedure is called parameter inference [13].

## Case studies

This section presents three Italian case studies in which geographic profiling, or *locus operandi*, was used to narrow the area for searching for a missing person. The data (the area of residence and all the places visited or frequented by the missing person) were mapped and processed on an open-source GIS platform—QGIS [37]. In these cases, the disappearance occurred during the COVID-19 pandemic lockdown in Italy. The lockdown restrictions meant that only the police were able to operate in the field. Because they were physically and exclusively limited, the searches became slower and more complex. The searches entailed a remote analysis of the *locus operandi* of the missing persons. Although the outcome of the geographic profiling was accurate and precise, the missing persons were found dead.

For reasons of privacy, the names of the missing subjects, geographical details of the areas of disappearance, and precise dates are not reported. The abbreviations MP1 (Missing Person 1, etc.), MP2, and MP3 have been used, with the province of origin and the month and year of disappearance.

### Case 1

In March 2020, MP1’s mother reported that her daughter had disappeared the previous day from their home in the province of Pisa. The 40-year-old daughter had a history of mental disorders and had left home without her mobile phone or documents, wearing pyjamas and a pair of slippers, in a state of evident agitation pertaining to the COVID-19 health emergency. The mother was aware of the direction initially taken and some places that her daughter frequented.

**Figure 2.**
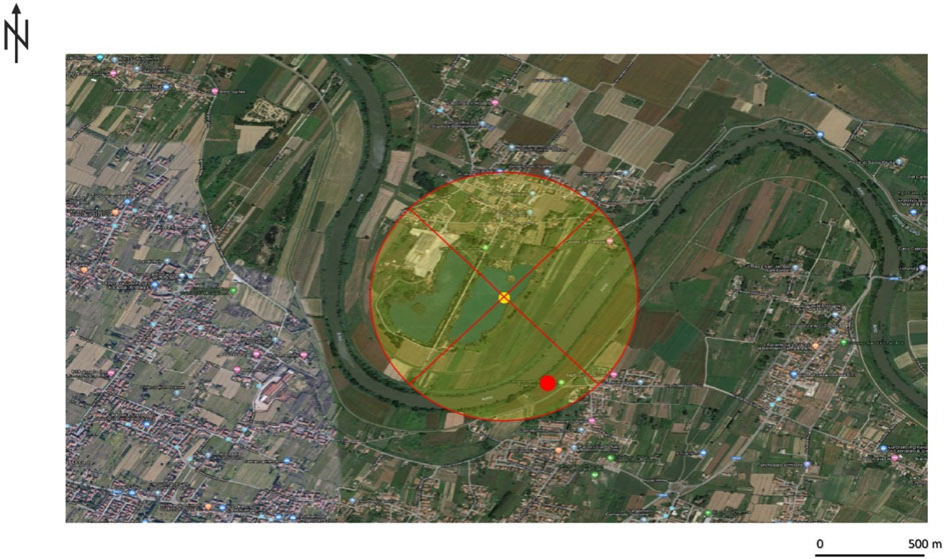
The result of the geographic profiling investigation in Case 1. The yellow dot indicates the centre of gravity of the search area of *r* = 700 m. The person was found lifeless at the point indicated by the red dot.

Based on the collection of geographical data provided by the mother and the presence of some water sources, it was possible to reconstruct a geographic profile of the disappearance to delimit the search area and assist the police. Unfortunately, the search was slowed down by the lockdown restrictions. However, the search had started and some areas had already been eliminated. The geographical data on the GIS platform was then analysed, to better define the superposition of Thiessen and Voronoi (Algorithm ID: qgis: voronoipolygons) with Delaunay (Algorithm ID: qgis: delaunaytriangulation), using the *Centroid* geometric algorithm (Algorithm ID: qgis: pointonsurface). This made it possible to identify a precise centre of gravity around which a buffer of radius *r* = 700 m was drawn, indicating where the search could be intensified (Figure 2). The lifeless body was later found within the highlighted area.

### Case 2

At the beginning of April 2020, an 80-year-old was declared missing by his son in the province of Brescia, when the father failed to return home one evening. Dressed in a sweater, sweatpants, and a pair of shoes, he left the house without saying anything or reporting where he was going. MP2 suffered from amnesia and left without his wallet, identification documents, or mobile phone. According to the son, the father was familiar with the places surrounding the house and had some favourite areas that he frequented.

Based on the geographical indications, it was possible to create the MP2's *locus operandi* and delimit a specific area in which to focus the search efforts. As in Case 1, the search in this case also proceeded slowly because of the COVID-19 health emergency. Therefore, profiling was made available to the police remotely.

**Figure 3.**
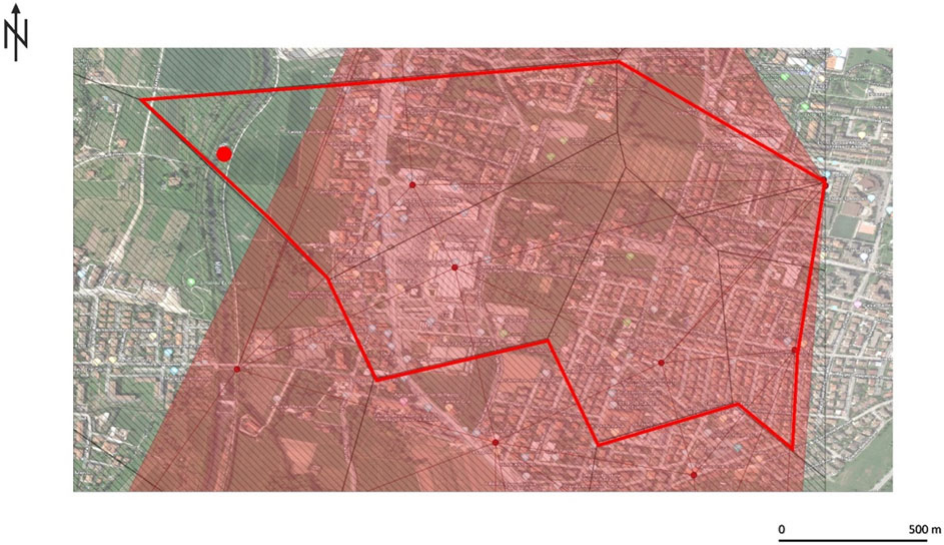
The result of the geographic profiling investigation in Case 2. The area enclosed by red lines indicates the region of interest for searching the missing person, found lifeless at the point indicated by the bright red dot.

The geographical data were analysed on the GIS platform by superimposing Thiessen and Voronoi (Algorithm ID: qgis: voronoipolygons) with Delaunay (Algorithm ID: qgis: delaunaytriangulation), thereby defining an area in which the search could be intensified (Figure 3). The lifeless body was later found within the highlighted area.

### Case 3

In early May 2020, the relatives of MP3 reported the disappearance of their 50-year-old relative in the province of Lucca. On the day of the disappearance, MP3 had dressed normally, had not carried his documents or telephone, and had been sighted in a specific part of the town, suffering from breathing difficulties. Later that day, he was seen riding his bicycle and disappeared soon after. It was also reported that he was a heavy drinker.

The relatives drew up a very accurate list of the various places frequented by MP3 both by bicycle and on foot. MP3 was very popular and well-known in the town but still vanished. Although the lockdown restrictions were easing gradually at that time, travelling was still largely prohibited. As a result, the search proceeded slowly, and the profiling of the *locus operandi* was drafted remotely.

**Figure 4.**
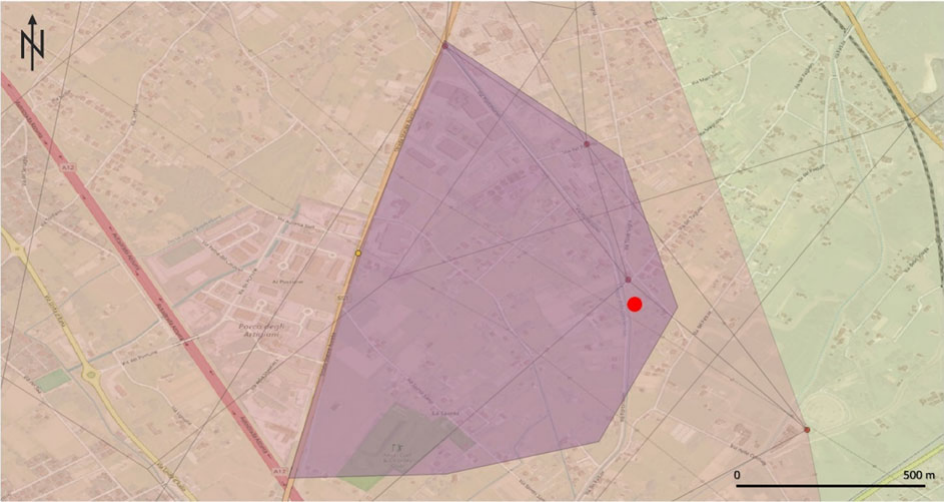
The result of the geographic profiling investigation in Case 3. The purple area indicates the region of interest for searching the missing person, found lifeless at the point indicated by the red dot.

In this case, as before, we analysed the geographical data on the GIS platform by superimposing Thiessen and Voronoi (Algorithm ID: qgis: voronoipolygons) with Delaunay (Algorithm ID: qgis: delaunaytriangulation), thereby defining an area where the search should be intensified (Figure 4). The lifeless body was later found within the highlighted area.

## Discussion and conclusions

In case of missing persons who are not related to a direct and evident crime, the possibility of using a geographic profiling approach can help law enforcement agencies in complex scenarios such as those illustrated in the previous section. Superimposing computational layers, together with the information derived from the adapted Rossmo formula, within a GIS platform creates a defined search area from which the police can start. Geographic profiling, or *locus operandi*, applied to disappearances should not be considered an additional resource, but an innovative and specific tool that revolutionises the method of finding missing persons and reduces the cost, risk, and stress to both human resources.

Although most disappearances follow a logical and specific pattern, geographic profiling does not take account of some subjective factors. The dynamics of the disappearance, like the chosen places, may have no logical model and may be dictated by sudden and irrational reactions. However, intelligent and automated geographic profiling models are necessary, and even fundamental, in improving the research techniques and reducing the number of people who are still missing in Italy and around the world. The geographical data that can be extracted from the reports of a disappearance constitute a desirable form of big data, which could be used to perform intelligent and automated spatial analyses. Nowadays, there is a concrete possibility of implementing this approach as an artificial intelligence (AI) procedure, whereby the law enforcement agencies can produce the geographic profile automatically. The feasibility of using AI is increased with GIS, and various results have been produced that support this claim [1, 38, 39]. The concrete results obtained by the authors of this article have shown how this approach can lead to the automation of the process, with the construction and implementation of a GIS artifact. This produces visualisations and statistical results to develop intelligent geographic profiling that supports predictive analysis of an area where a missing person may be found. A research information system prototype guides the investigation. It uses the disappearance as a dependent variable and a body of geographic information on frequented places—accompanied by geolocation and remote sensing data (such as satellite or camera images)—as the independent variable. This spatial information processing as a predictive variable of disappearance is vital in conducting a solid and secure analysis, particularly for Italian scenarios, in which we have several cases of missing persons.
